# A safety study of intraoperative radiation therapy following stereotactic body radiation therapy and multi-agent chemotherapy in the treatment of localized pancreatic adenocarcinoma: study protocol of a phase I trial

**DOI:** 10.1186/s13014-022-02145-9

**Published:** 2022-10-28

**Authors:** Abhinav V. Reddy, Colin S. Hill, Lei Zheng, Jin He, Amol K. Narang

**Affiliations:** 1 Sidney Kimmel Cancer Center, Department of Radiation Oncology and Molecular Radiation Sciences, Johns Hopkins University School of Medicine, 401 N Broadway, Baltimore, MD 21231 USA; 2 Sidney Kimmel Cancer Center, Department of Oncology, Johns Hopkins University School of Medicine, 401 N Broadway, Baltimore, MD 21231 USA; 3 Sidney Kimmel Cancer Center, Department of Surgery, Johns Hopkins University School of Medicine, 401 N Broadway, Baltimore, MD 21231 USA

**Keywords:** Intraoperative radiation therapy, Stereotactic body radiation therapy, Pancreatic cancer, Mesopancreas, Triangle volume, Dose escalation

## Abstract

**Background:**

Localized pancreatic adenocarcinoma carries a poor prognosis even after aggressive therapy. Up to 40% of patients may develop locoregional disease as the first site of failure. As such, there may be a role for intensification of local therapy such as radiation therapy. Radiation dose escalation for pancreatic cancer is limited by proximity of the tumor to the duodenum. However, the duodenum is removed during Whipple procedure, allowing the opportunity to dose escalate with intraoperative radiation therapy (IORT). Although prior studies have shown potential benefit of IORT in pancreatic cancer, these studies did not utilize ablative doses (biologically effective dose [BED_10_] > 100 Gy). Furthermore, the optimal radiation target volume in this setting is unclear. There has been increased interest in a “Triangle Volume” (TV), bordered by the celiac axis, superior mesenteric artery, common hepatic artery, portal vein, and superior mesenteric vein. Dissection of this area, has been advocated for by surgeons from Heidelberg as it contains extra-pancreatic perineural and lymphatic tracts, which may harbor microscopic disease at risk of mediating local failure. Interestingly, a recent analysis from our institution indicated that nearly all local failures occur in the TV. Therefore, the purpose of this protocol is to evaluate the safety of delivering an ablative radiation dose to the TV with IORT following neoadjuvant chemotherapy and stereotactic body radiation therapy (SBRT).

**Methods:**

Patients with non-metastatic pancreatic adenocarcinoma centered in the head or neck of the pancreas will be enrolled. Following treatment with multi-agent neoadjuvant chemotherapy, patients will undergo SBRT (40 Gy/5 fractions) followed by IORT (15 Gy/1 fraction) to the TV during the Whipple procedure. The primary objective is acute (< 90 days) toxicity after IORT measured by Clavien-Dindo classification. Secondary objectives include late (> 90 days) toxicity after IORT measured by Clavien-Dindo classification, overall survival, local progression-free survival, distant metastasis-free survival, and progression-free survival.

**Discussion:**

If the results show that delivering an ablative radiation dose to the TV with IORT after neoadjuvant chemotherapy and SBRT is safe and feasible, it warrants further investigation in a phase II trial to evaluate efficacy of this approach.

*Trial Registration* This study was registered at ClinicalTrials.gov on 12/2/2021 (NCT05141513). https://clinicaltrials.gov/ct2/show/NCT05141513

## Background

Pancreatic adenocarcinoma (PDAC) is the third most common cause of cancer related deaths in the United States, responsible for over 48,000 deaths each year [1]. At time of diagnosis, approximately 10% are resectable, 40% are borderline resectable (BRPC) or locally advanced (LAPC), and the remaining 50% are metastatic. Treatment of localized disease usually involves a combination of multi-agent chemotherapy, radiation therapy, and surgery [2]. Unfortunately, even with aggressive therapy, prognosis is poor with 5-year overall survival (OS) of less than 15% [3].

Improvements in chemotherapy and radiation therapy have increased the proportion of patients with non-metastatic disease who can undergo surgery [4, 5]. However, after surgery, while distant failure predominates, a not insignificant portion of patients (30–40%) develop local recurrence [6, 7]. In fact, one report demonstrated that 30% of all pancreatic cancer patients die from locally destructive disease [8]. This suggests that there may be a role for intensification of local therapies such as radiation. However, dose escalation of radiation therapy in this context is limited by proximity to gastrointestinal structures and in particularly the duodenum. Given that the duodenum is resected during Whipple surgery, intra-operative radiation therapy (IORT) provides a unique opportunity to intensify radiation delivery for pancreatic cancer and further escalate the radiation dose delivered beyond what is feasible with external beam radiation therapy alone, even with modern technologies such as stereotactic body radiation therapy (SBRT). Additionally, IORT allows for shielding of other normal organs, direct visualization of the target, and elimination of positional uncertainties.

Several studies have investigated the role of IORT for localized pancreatic cancer and have suggested that the addition of IORT may improve local control without additional acute or late toxicity [9–11]. Keane et al. evaluated a group of BRPC/LAPC who underwent neoadjuvant chemotherapy and chemoradiation, followed by surgical resection with or without IORT [10]. The addition of IORT led to improved median survival (35.1 vs 24.5 months, *p* = nss) with no differences in intraoperative blood loss, 90-day readmission rates, or post-operative complications between the two groups. A more recent study by Sekigami et al. showed that IORT after neoadjuvant chemotherapy and chemoradiation in BRPC/LAPC mitigates the poor prognosis of R1 resection when compared to no IORT. There was no difference in 90-day mortality rate or 30-day major complication rate (IORT: 17% vs no IORT: 22%, *p* = 0.477) [11].

Although the aforementioned retrospective reports suggest the efficacy and safety of IORT, there are few prospective studies [12]. Furthermore, prior studies have used conventional dose fractionation schemes, with a total radiation dose (external beam radiation therapy + IORT) less than what is considered ablative (Biologically effective dose [BED_10_] > 100 Gy). Additionally, many of these studies were performed prior to the incorporation of FOLFIRINOX chemotherapy, which has been shown to improve OS and disease-free survival. Finally, these studies limited IORT fields to the specific vascular margins at risk as opposed to a larger peri-pancreatic target encompassing perineural tracts and lymphovascular channels at risk of microscopic residual disease after resection, which may drive local failure in this population [13].

The optimal radiation volume in the treatment of localized pancreatic cancer is currently unknown. However, there is growing interest among surgeons in the dissection of the space between the celiac artery (CA), superior mesenteric artery (SMA), common hepatic artery (CHA), portal vein (PV), and superior mesenteric vein (SMV) [13, 14]. This region is thought contain extra-pancreatic perineural and lymphatic tracts, which can harbor microscopic disease, and therefore, mediate locoregional relapse. Surgeons from Heidelburg are proponents of the “Triangle Operation”, which involves extended dissection of this space [14]. In fact, our group previously performed an analysis in which the location of local failures was mapped [15], and this analysis showed that local failures are nearly universally located within this space, which we will hereto forward refer to as the “Triangle Volume (TV).”

As such, the goal of this protocol is to prospectively explore the safety and feasibility of implementing IORT in patients with localized pancreatic cancer undergoing surgical resection after pre-operative chemotherapy and SBRT, with the goal of safely reaching ablative radiation doses to the TV when combined with pre-operative SBRT. At the conclusion of this study, we will compare the safety outcomes to prior institutional data [16]. If deemed safe and feasible, we will plan to open a phase II trial to formally evaluate the efficacy of this approach with the goal of improving local control for this disease.

## Methods/design

### Objectives

The primary objective is to evaluate acute post-operative toxicity (< 90 days) of IORT targeted to the TV in patients with non-metastatic pancreatic adenocarcinoma undergoing surgical resection after neoadjuvant multi-agent chemotherapy and SBRT.

The secondary objectives are as follows: 1.To evaluate late toxicity (> 90 days) of IORT targeted to the TV in patients with non-metastatic PDAC undergoing surgical resection after neoadjuvant multi-agent chemotherapy and SBRT, 2. Estimate median local progression-free survival (LPFS) from time of IORT, 3. Estimate median overall survival (OS) from time of IORT, 4. Estimate median distant metastasis-free survival (DMFS) from time of IORT and 5. Estimate median progression-free survival (PFS) from time of IORT.

### Study design

**Fig. 1:**
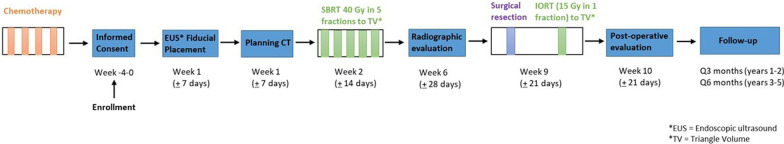
Study protocol schema

This is a prospective, single institution, single arm safety study to evaluate the safety and feasibility of implementing IORT in patients with non-metastatic PDAC centered in the head or neck of the pancreas who have been treated with neoadjuvant chemotherapy and SBRT and who are undergoing Whipple procedure. All patients will be discussed in our institutional pancreatic multi-disciplinary clinic. Patients with localized PDAC will be considered for enrolling in the study. Patients will be formally enrolled after completion of chemotherapy and just prior to SBRT. This would allow us to assess treatment response with imaging and exclude patients who developed distant disease during chemotherapy. Table 1 shows the protocol calendar and Fig. 1 displays the protocol schema.

**Table 1:** Study calendar

Week# (± days)	− 4–0	1 (± 7 days)	2 (± 14 days)	6 (± 28 days)	9 (± 21 days)	10 (± 21 days)	Q3mos (years 1–2) Q6mos (years 3–5)
Interventions/Procedures							
EUS Fiducial placement		X					
CT Simulation		X					
SBRT × 5 days			X				
Surgery					X		
IORT (15 Gy/1 fraction)					X		
Informed Consent	X						
Inclusion/Exclusion Criteria	X						
Demographics	X						
Medical History	X						X
Medications	X						X
Vital Signs and Pulse Oximetry	X						X
Physical exam	X						X
Height	X						
Weight	X						X
KPS	X						X
ECOG status	X						X
Pre-operative evaluation by surgery				X			
Toxicity Assessment^1^						X^^^	X
*Tests*							
Planning CT Abdomen^2^						X	
Radiographic evaluation^3^				X			X

*EUS* Endoscopic ultrasound, *CT* Computed tomography, *SBRT* Stereotactic body radiation therapy, *IORT* Intraoperative radiation therapy, *KPS* Karnofsky Performance Status, *ECOG* Eastern Cooperative Oncology Group, *CTCAE* Common Terminology Criteria for Adverse Events

^1^CTCAE v5.0 and Clavien-Dindo classification

^2^In Radiation Oncology department

^3^Diagnostic CT Abdomen

^^^Inpatient evaluation: post-operative hospitalization

### Ethics

This study was conducted in accordance with the Declaration of Helsinki (as revised in 2013). The study was approved by the institutional review board (IRB) of Johns Hopkins University (IRB00294801). All subjects or an authorized representative, will be informed of the nature of the study and will provide written informed consent, approved by the IRB of Johns Hopkins University. All patients enrolled on this protocol will undergo discussions with health care providers regarding risks, benefits, and details of each intervention. This study has been registered at ClinicalTrial.gov (NCT05141513).

### Interventions

#### Chemotherapy

All patients will be treated upfront multi-agent chemotherapy, with the exact regimen and duration at the discretion of the treating medical oncologist. During chemotherapy, patients will generally have pancreatic protocol imaging approximately every 2 months to assess treatment response.

#### Stereotactic body radiation therapy

After completion of chemotherapy, patients will be planned for SBRT to 40 Gy in 5 fractions. This dose and fractionation is recommended by the Australasian Gastrointestinal Trials and Trans-Tasman Radiation Oncology Group [17]. Prior to SBRT, all patients will have ultrasound guided endoscopic gold or platinum fiducial placement for the purpose of daily image guidance. At time of simulation, patients will be positioned supine with arms above head in a Vac-Lok (CIVCO Medical Solutions, Coralville, IA, USA) for immobilization. Thin-sliced computed tomography (CT) scans with intravenous contrast will be obtained for radiation treatment planning. Motion management with active breathing control (ABC, Elekta, Stockholm, Sweden) will be performed. Patients who cannot tolerate breath-hold will be treated under free-breathing conditions, with an internal target volume (ITV) generated from the peak inspiratory and expiratory phases from a 4-dimensional CT scan. The clinical target volume (CTV) and organs at risk will be contoured using Pinnacle treatment planning system (Phillips Radiation Oncology Systems, Fitchburg, WI). The CTV will include gross disease as well as the TV, which includes the space between the CA, SMA, CHA, PV, and SMV. The planning target volume (PTV) will be generated by adding a patient-specific margin to the CTV based on assessment of variability in fiducial positioning between multiple breath-hold scans acquired at simulation (usually on the order of 2-4 mm) followed by an additional 2 mm isotropic expansion. For free-breathing cases, the PTV will be generated by adding a 2 mm isotropic expansion to the iCTV. Daily image guidance with pre-treatment and intrafractional cone-beam CT scans will be performed to ensure proper setup. Patients will be aligned to spine and then shifted to align to fiducials.

#### Surgery

Approximately 1–8 weeks after completion of SBRT, patients will have restaging imaging. The decision for surgical exploration will be at the discretion of the surgical oncologist. Surgery may take place anywhere from 2 and 10 weeks after completion of SBRT. Intraoperatively, a photo will be taken to document dissection of the TV.

#### Intraoperative radiation therapy

Intraoperative radiation therapy will be delivered with high-dose-rate (HDR) brachytherapy using a Freiburg flap applicator. This approach was chosen because the Freiburg flap can be placed to ensure proper contact with key vasculature that define the borders of the TV and the ability to space the applicator into the area at risk. A Freiburg flap that corresponds in size to the TV will be constructed. A radiation plan will be generated in conjunction with the medical physics team to optimally target the TV with 15 Gy, prescribed to the surface of the volume. Catheters will be threaded through the Freiburg Flap, secured in place, and numbered**.** The Freiburg Flap will then be positioned into the TV. Lead shields will be placed between the Freiburg Flap and normal organs at risk, including stomach, remaining small and large bowel, ureters, etc. The catheters will subsequently be connected to the afterloader system that houses the Ir-192 source. A “dummy” run will be performed to ensure no kinks in the system. The operative room will subsequently be cleared of personnel, and remote monitoring from the control room will be confirmed. A timeout will subsequently be performed, after which treatment will commence to deliver 15 Gy in 1 fraction. Upon completion of treatment, the room will be checked for any radio-activity, after which personnel can subsequently re-enter the room. At this point, surgical clips will be placed along the vessels that border the flap to confirm dosimetry on post-operative imaging. The Freiburg Flap and any lead will be removed. During the post-operative hospital stay, patients will undergo a CT Abdomen in the radiation oncology department to confirm dosimetry and ensure appropriate radiation coverage.

#### Rational for radiation dose

A total radiation dose of 109.5 Gy BED_10_ will be delivered, with SBRT in 40 Gy in 5 fractions (BED_10_ = 72 Gy) and IORT in 15 Gy in 1 fraction (BED_10_ = 37.5 Gy). This dose was chosen because BED_10_ > 100 Gy has been associated with excellent local control in both pancreatic cancer and other solid tumors [18–20]. Stereotactic body radiation therapy to 40 Gy in 5 fractions is recommended by the Australasian Gastrointestinal Trials and Trans-Tasman Radiation Oncology Group [17]. Intraoperative radiation therapy to 15 gy in 1 fraction is a commonly used dose for pancreatic cancer with a range of 12.5–20 Gy in 1 fraction [10–12].

#### Follow-up

Patients will follow-up in clinic every 3 months with imaging for the first 2 years post-surgery and then every 6 months with imaging for years 3–5 post-surgery. During each follow-up visit, patients will undergo a physical exam, assessment of performance status, and have routine blood work including cancer antigen 19–9. Additionally, chemotherapy-induced and radiation-induced toxicity will be assessed using the Common Terminology Criteria for Adverse Events (CTCAE) criteria (v5.0). Surgical- toxicity will be assessed using Clavien-Dindo classification. Toxicity will be evaluated at each follow-up visit. Disease progression and response will be assessed using Response Evaluation Criteria in Solid Tumors (RECIST) criteria (v1.1).

### Inclusion criteria

Subjects must meet all of the following criteria to be eligible for participation in the study:

1. Age ≥ 18 years old
2. Resectable/BRPC/LAPC as defined by National Comprehensive Cancer Network guidelines [2] confirmed via CT, endoscopic ultrasound, or other imaging modalities.
3. Eastern Cooperative Oncology Group performance status 0–2
4. Subject or authorized representative, has been informed of the nature of the study and has provided written informed consent, approved by the appopriate Institutional Review Board of Johns Hopkins Hospital
5. Upfront treatment with multi-agent chemotherapy
6. Tumor location in the pancreatic head or neck that would be technically amenable to Whipple resection
7. Candidate for surgical exploration at Johns Hopkins Hospital
8. Candidate for SBRT at Johns Hopkins Hospital

### Exclusion criteria

Sujects who meet any of the following criteria are not eligible for participation in the study:

1. Previous thoracic/abdominal radiation therapy
2. Unable to receive SBRT at Johns Hopkins Hospital
3. Duodenal invasion detected on imaging which would exclude candidacy for SBRT
4. Tumor located in pancreatic body or tail
5. Medical or technical contraindications to a Whipple procedure
6. Evidence of disease not localized to the pancreas
7. Any arterial reconstruction during surgery
8. Currently enrolled in another investigational drug or device trial that clinically interferes with this study
9. Unable to comply with study requirements or follow-up schedule
10. Women of child bearing potential or sexually active fertile men with partners who are women of child bearing potenital who are unwilling or unable to use an acceptable method to avoid pregnancy for the entire study

### Statistical analysis and sample size

Descriptive statistics will be utilized to record patient demographic, disease, and treatment characteristics including age, sex, race, disease extent, chemotherapy, radiation therapy, and type of surgery. Time to event outcomes such as OS, LPFS, DMFS, and PFS will be analyzed using Kaplan–Meier method. Our institution’s data and experience suggest that 15% of patients will not be surgically explored due to development of radiographic metastatic disease or too locally extensive disease after SBRT. An additional 15% will require unexpected arterial reconstruction (exclusion criteria). Therefore, 25 patients will be enrolled with the expectation that 20/25 patients will undergo surgical exploration and IORT. A sample size of 20 patients was chosen because this will enable safety evaluation using a closely monitored Bayesian stopping rule and is also recommended by the literature [21, 22]. There will not be any interim data analysis. Data analysis will occur once the study has been completed.

### Study endpoints

Primary endpoint: post-operative complications in first 90 days following IORT will be recorded as a binary variable (≥ Clavien-Dindo grade IIIa or < Clavien-Dindo grade IIIa). Clavien-Dindo classification is displayed in Table 2 [23].

**Table 2:** Clavien-Dindo classification

Grade I	Any deviation from the normal postoperative course without the need for pharmacological treatment or surgical, endoscopic, and radiological interventions. Allowed therapeutic regimens are: drugs as antiemetics, antipyretics, analgesics, diuretics, electrolytes, and physiotherapy. This grade also includes wound infections opened at the bedside
Grade II	Requiring pharmacological treatment with drugs other than such allowed for grade I complications Blood transfusions and total parenteral nutrition are also included
Grade III	Requiring surgical, endoscopic or radiological intervention
IIIa	Intervention not under general anesthesia
IIIb	Intervention under general anesthesia
Grade IV	Life-threatening complication (including CNS complications)* requiring IC/ICU management
IVa	Single organ dysfunction (including dialysis)
IVb	Multi-organ dysfunction
Grade V	Death of a patient

^*^Brain hemorrhage, ischemic stroke, subarachnoid bleeding, but excluding transient ischemic attacks

*CNS* Central nervous system, *IC* Intermediate care, *ICU* Intensive care unit

Secondary endpoints are as follows: 1. Post-operative complications after 90 days following IORT will be recorded as a binary variable (≥ Clavien-Dindo grade IIIa or < Clavien-Dindo grade IIIa), 2.Local progression-free will be recorded as time from IORT to first occurrence of locoregional or last known negative imaging. Locoregional failure will include disease recurrence occurring within the surgical bed, extra-pancreatic perineural tracts, regional nodal basins, or the triangle volume, 3. Overall survival will be recorded as the time from IORT to death or last known clinic follow-up, 4. Distant metastasis-free survival will be measured from time of IORT to the development of distant progression or last known negative imaging. 5. Progression-free survival will be measured as the interval from the end of IORT to the time of the first radiographic evidence of any failure, death, or last known negative imaging.

### Safety stopping rules

To minimize the risks of IORT, safety will be monitored by a Bayesian stopping rule for the rate of adverse events of interest. Previous experience demonstrated that the incidence of 90-day Clavien-Dindo Grade ≥ IIIa is 25% [23]. Safety will be monitored continuously for 20 patients through day 90.

Adverse events (AE) will be monitored continuously for all patients. A Bayesian safety monitoring rule will be used to evaluate the rate of the AE of interest continuously, from the 3rd evaluable patient, and will suspend accrual at any point if there is sufficient evidence of excessive toxicity. Specifically, the Bayesian toxicity monitoring rule will suspend accrual anytime if the posterior probability of AE of interest being larger than 40%, is 70% or higher. We assume a priori that the experimental regimens has an average risk of 25% and there is a 25% chance that the risk will be 40% or higher. Table 3 summarizes the continuous stopping rule for the 20 evaluable patients for each regimen. For example, if 2 patients out of the first 3 evaluable patients experience adverse events of interest, we will stop accrual. Furthermore, to cautiously assess the toxicity profiles of experimental regimens in the event that accrual is relatively “fast” comparing to the safety assessment duration, the accruals of each cohort will be suspended when 6 eligible patients have been accrued to allow the continuous safety evaluations to be done among the first 6 evaluable patients before further accruals. The accrual would resume only if the Bayesian stopping rule utilized is not met. At any time if the stopping criterion is met, accrual to the trial will be temporarily suspended and the principle investigators and study team will review the toxicity data and recommend either modification or termination of the trial.

**Table 3:** Stopping rule for safety

Number patients with toxicity of interest	2	3	4	5	6	7	8	9	10
Total number patients treated	3	4–5	6–7	8–9	10–12	13–14	15–16	17–19	20

Table 4 summarizes the operating characteristics based on 5,000 simulations with 20 evaluable patients in terms of how frequent the study would stop based on the stopping rule under different hypothetical toxicity rates, as well as the average sample sizes.

**Table 4:** Operating characteristics of the stopping rule for safety

Underlying risk	0.25	0.30	0.35	0.40
% of time study stops	21.6%	32.1%	45%	58.5%
Expected sample size	16.7	15.2	13.4	11.7

## Discussion

Although distant progression is most common after primary resection of pancreatic cancer, locoregional relapse can be common, with up to 40% patients developing locoregional disease as the first site of failure [24, 25]. Uncontrolled locoregional progression can negatively impact morbidity and mortality outcomes [26]. Cardillo et al. demonstrated that the uncontrolled locoregional disease was the most common cause of hospitalization in pancreatic cancer patients [26]. Therefore, intensification of local therapy, such as dose-escalated radiation, is warranted. Dose escalation in pancreatic cancer is limited by proximity of the tumor to the duodenum. However, the duodenum is removed during Whipple procedure, allowing opportunity for dose escalation using IORT.

There is debate regarding the appropriate treatment volumes for localized pancreatic cancer in the pre-operative and/or definitive setting. While consensus guidelines recommend treatment of gross disease alone, others suggest that treatment of elective nodal regions in addition to gross disease may provide benefit [27–30]. Surgeons from Heidelberg have advocated for extended dissection of the space between the CA, SMA, CHA, PV, and SMV, termed the “Triangle Operation” [14]. This region, which we call the “Triangle volume”, is thought to contain extrapancreatic perineural tracts and lymphatic channels, which may harbor microscopic disease and mediate locoregional relapse [13]. In fact, a recent analysis from our institution [15] demonstrated that nearly all local failures occur within this TV.

This protocol will evaluate the safety and feasibility of delivering an ablative dose of radiation to the TV using IORT (15 Gy/1 fraction) after neoadjuvant chemotherapy and SBRT (40 Gy/5 fractions) in patients undergoing Whipple procedure. A total BED_10_ of 109.5 Gy will be delivered to the TV. If the results show that dose escalation with IORT is safe and feasible, it warrants further investigation in a phase II setting to evaluate efficacy of this approach.

## Data Availability

Not applicable.

## Abbreviations

PDAC: Pancreatic adenocarcinoma
BRPC: Borderline resectable pancreatic cancer
LAPC: Locally advanced pancreatic cancer:
IORT: Intraoperative radiation therapy
SBRT: Stereotactic body radiation therapy
BED_10_: Biologically effective dose
CA: Celiac axis
SMA: Superior mesenteric artery
CHA: Common hepatic artery
PV: Portal vein
SMV: Superior mesenteric vein
TV: Triangle volume
OS: Overall survival
LPFS: Local progression-free survival
DMFS: Distant metastasis-free survival
PFS: Progression-free survival
IRB: Institutional review board
CT: Computed tomography
ITV: Internal target volume
CTV: Clinical target volume
PTV: Planning target volume
HDR: High-dose-rate
CTCAE: Common terminology criteria for adverse events
RECIST: Response evaluation criteria in solid tumors
AE: Adverse events
